# Treatment Adherence to Injectable Treatments in Pediatric Growth Hormone Deficiency Compared With Injectable Treatments in Other Chronic Pediatric Conditions: A Systematic Literature Review

**DOI:** 10.3389/fendo.2022.795224

**Published:** 2022-03-01

**Authors:** Roy Gomez, S. Faisal Ahmed, Mohamad Maghnie, Dejun Li, Toshiaki Tanaka, Bradley S. Miller

**Affiliations:** ^1^ Global Medical Affairs, Pfizer, Ixelles, Belgium; ^2^ Developmental Endocrinology Research Group, School of Medicine, University of Glasgow, Glasgow, United Kingdom; ^3^ Clinica Pediatrica, IRCCS Istituto Giannina Gaslini, Genoa, Italy; ^4^ Department of Neurosciences, Rehabilitation, Ophthalmology, Genetics, Maternal and Child Health (DINOGMI), University of Genoa, Genoa, Italy; ^5^ Center for Prenatal Diagnosis and Reproductive Medicine, The First Hospital of Jilin University, Changchun, China; ^6^ Tanaka Growth Clinic, Tokyo, Japan; ^7^ Pediatric Endocrinology Division, University of Minnesota Masonic Children’s Hospital, Minneapolis, MN, United States

**Keywords:** adherence, injection, growth hormone, growth hormone deficiency, pediatric, systematic literature review

## Abstract

**Background:**

Pediatric patients with growth hormone deficiency (GHD) are currently treated with daily injections of recombinant human growth hormone (rhGH) to promote linear growth and enable attainment of normal adult height. One of the main reasons for suboptimal growth during rhGH therapy is non-adherence to treatment. The objective of this systematic literature review was to examine the recent literature on pediatric adherence to injectable treatments for chronic conditions (focusing on rhGH) to characterize levels of adherence and identify the factors/barriers associated with adherence.

**Methods:**

The Embase and MEDLINE databases (January 2015–October 2020) were searched to identify publications describing studies of pediatric patients (aged ≤17 years) with GHD and other chronic conditions requiring daily or weekly injectable treatments; a similar targeted search of Chinese literature was also performed. Adherence data were extracted from the included studies and summarized. Risk of bias was determined using the Cochrane Risk of Bias tool 2 or the Newcastle-Ottawa Scale.

**Results:**

A total of 23 publications were included, with all publications except for one (multiple sclerosis) focused on pediatric GHD studies: there were two clinical trials, 18 observational studies and three survey studies. Study sample sizes ranged from 30 to 13,553 patients (median: 95 patients). The definition of adherence varied between studies and included mean adherence rate, median adherence rate, and the percentage of patients within pre-specified adherence categories. Of the publications assessing adherence to daily rhGH, 11 studies reported 12-month mean adherence rate (range: 73.3%– 95.3%) and eight studies reported median adherence (range: 91%– 99.2%). The barriers to treatment adherence identified included self-administration, increased administration frequency, age (adolescence), longer treatment duration, device design, and insufficient family education, awareness, and/or engagement. Recommendations for increasing adherence included using adherence reminder tools, increasing patient engagement/education, and improving injection device design and drug product.

**Conclusions:**

Adherence to rhGH treatment was high (>80%) for many studies, though comparability between studies was limited given the substantial heterogeneity in the way adherence was defined, measured, and reported. To address this heterogeneity, we recommend standardizing how adherence is defined and reported and encourage the use of standardized study designs and outcome measures.

## Introduction

Growth hormone deficiency (GHD) is characterized by insufficient secretion of human growth hormone (hGH) from the pituitary gland and low serum concentrations of insulin-like growth factor-1 (IGF-1). In children with GHD, growth of skeletal and muscle mass is reduced, resulting in delayed puberty and height below the normal range. Treatment for GHD consists of recombinant hGH (rhGH), which has been shown to promote linear growth in children with GHD, enabling them to achieve normal height in adulthood (1). Currently, most forms of rhGH require administration *via* daily subcutaneous (SC) injections. Despite the efficacy of rhGH and an increased range of injection devices available to patients (1, 2), treatment results can be suboptimal (3–5). The failure of patients with GHD to reach target adult height despite receiving rhGH treatment remains a prevalent outcome (1, 6).

Although the reasons for suboptimal outcomes following rhGH treatment are likely to be multifactorial, it is widely acknowledged that non-adherence to daily injections plays an important role. A study of 217 GHD patients from six pediatric endocrinology clinics in Turkey found that height velocity (HV) and HV standard deviation scores (SDS) in patients with optimal adherence to rhGH therapy were higher than in patients with suboptimal adherence, as were levels of IGF-1, which was correlated with HV and HV SDS (7). Two systematic literature reviews (SLRs) in the last decade examined the paradigm surrounding non-adherence (1, 8). The earlier SLR (1) found that 5–82% of patients miss at least some rhGH doses. Fisher et al. identified the following injection-related factors as being associated with non-adherence: perceived difficulty of injections; lack of choice of injection device; short duration of prescriptions; and discomfort (1). The later SLR by Graham et al. (8) reported that 7–71% of pediatric GHD patients were non-adherent. Of the 22 factors identified as being associated with non-adherence, those related to daily injections included: injection-related pain and discomfort; poor administration technique; forgetting injections; disruption in supply of injections due to short duration of prescriptions; and being away from home (8).

In order to better characterize the levels of adherence and the factors associated with adherence in pediatric patients with GHD, a review of the literature was performed to consider the most recent evidence from the field. To capture insights beyond the existing literature in this field (1, 8), the review also considered evidence from other pediatric therapeutic areas that require regular (daily or weekly) self- or caregiver-administered injectable treatments. These included multiple sclerosis (MS), juvenile idiopathic arthritis (JIA), and inflammatory bowel disease (IBD). Specifically, this review investigates the drivers and barriers to rhGH adherence, as well as recommendations and best practices that can be applied to improve adherence to rhGH.

The objectives of this SLR were to (i) summarize the recent literature on pediatric adherence to injectable treatments for chronic conditions, with a focus on rhGH, and (ii) identify factors associated with pediatric adherence/non-adherence to rhGH and other injectable treatments.

## Methods

This review was guided by the principles of the Interim Guidance from the Cochrane Rapid Reviews Methods Group (9) and guidance from the Centre for Reviews and Dissemination (10). The eligible study populations, interventions, comparators, outcomes, and study types (PICOS) are described in detail in **Table 1** and briefly below.

**Table 1:** Summary of eligibility criteria.

	Included	Excluded
**Population**	Children aged <18 years with GHD, an rhGH- indicated condition, or a chronic condition requiring daily or weekly self- or caregiver-administered injectable treatment (MS, JIA, IBD) Parents or caregivers of pediatric patients treated with regular injections for these conditions	Studies in adults or where outcomes of pediatric patients are not reported separately from those of adult patients
**Intervention**	rhGH or a self- or caregiver-administered injectable drug (SC or IM) indicated for ongoing daily or weekly treatment of chronic conditions (MS, JIA, IBD) in pediatric populations	Interventions that are not delivered by SC or IM injection (i.e., topical, oral, or infusion) Interventions that are not identified as SC or IM injection only and that could include other administration routes (e.g., “biologics” if this category includes infused or IV agents) (**Supplementary Table S1**)
**Comparator**	Any or none	Not applicable
**Outcomes**	Clearly identifiable/defined standardized measures (validated or non-validated) **AND** reported prevalence of adherence/non- adherence or compliance/non-compliance to injectable drugs, **OR** Explicitly identifiable and measured (a) barriers to adherence, **OR** (b) characteristics of patients, families/caregivers, providers, or institutions associated with adherence to prescribed treatment, **OR** (c) properties of the treatment, such as administration route or schedule, associated with adherence	Publications that (a) do not define how adherence was measured, or (b) do not report rate of adherence/non-adherence (e.g., discontinuation or persistence only would be excluded), or (c) do not report barriers/factors affecting adherence For inclusion, outcomes must be reported for SC or IM injectable drugs separately from infusion/IV, oral, or other routes of administration (**Supplementary Table S1**)
**Study design**	Observational studies (prospective or retrospective; including cohort studies, cross- sectional studies, or surveys) RCTs or non-RCTs (if reporting medication adherence or compliance)	Studies with non-empirical, theoretical, or narrative discussion of adherence and no quantitative measure of adherence Publications reporting methods or tool development, unless they report either a quantitative measure of adherence/non- adherence or factors associated with adherence Other study designs were not eligible (e.g., pre-clinical, case reports/studies reporting patient- level data only, economic studies, pooled data analyses, or meta-analyses) Systematic reviews published from 2015 onwards were not eligible for inclusion but were hand-searched for additional relevant references
**Limits**	Published in peer-reviewed journal from 2015 to 2020 Studies not published in English will be considered, with data extraction limited to English language elements and numerical data	Unpublished data and data from conference abstracts

GHD, growth hormone deficiency; IBD, inflammatory bowel disease; IM, intramuscular; IV, intravenous; JIA, juvenile idiopathic arthritis; MS, multiple sclerosis; RCT, randomized contrail trial; rhGH, recombinant growth hormone; SC, subcutaneous.

### Eligibility Criteria

Studies evaluating pediatric patients (ages 17 years and younger) with GHD and other chronic conditions (MS, JIA, or IBD) requiring daily or weekly injectable treatments were eligible for inclusion in this review. Studies that included young adults (e.g., ages 18–25 years) were eligible if they also included patients younger than 18 years of age, meaning that some patients in the transition period could be included. Studies reporting on self- or caregiver-administered rhGH or an injectable drug, *via* an SC route, for ongoing daily or weekly treatment of chronic conditions (MS, JIA, or IBD) in pediatric populations were eligible for inclusion. Studies with and without comparators were eligible.

Publications were eligible for inclusion if they reported one or more of the following outcomes: (i) clearly defined standardized measures of adherence/non-adherence or compliance/non-compliance AND reported prevalence of adherence/non-adherence or compliance/non-compliance to injectable drugs or (ii) identifiable and measured (a) barriers to adherence OR (b) characteristics of patients, families/caregivers, providers, or institutions associated with adherence/non-adherence to treatment OR (c) properties of the treatment associated with adherence/non-adherence. Observational studies (prospective or retrospective; including cohort studies, cross-sectional studies, or surveys), or randomized controlled trials (RCTs) or non-RCTs (if reporting medication adherence or compliance) were eligible for inclusion. Publications were required to have been published in peer-reviewed journals between 2015 and 2020.

### Study Selection

Searches were conducted in the following databases: Embase^®^ *via* Ovid and MEDLINE^®^ *via* Ovid. A detailed search strategy for each database is presented in **Supplementary Methods 1** and **Supplementary Tables S1, S2**. From the records identified in the database searches, one researcher (CR) excluded records that were irrelevant, which were then checked by a second researcher (KS). One researcher (KS) screened the titles and abstracts (Level 1) of the identified publications against the eligibility criteria. A second researcher (CR) screened 20% of the records to ensure agreement of screening decisions. The records identified in Level 1 were then subjected to full-text screening (Level 2) using the same process (initial screen by KS followed by validation of 20% of the records by CR). Screening discrepancies were discussed and resolved.

In addition to the Embase and MEDLINE searches, a targeted search of the Chinese literature was also conducted to identify additional publications on adherence to rhGH therapy in pediatric populations. An author fluent in Chinese (DL) conducted a literature search for publications in Chinese in the Wan Fang database (http://www.wanfangdata.com.cn/index.html).

### Data Extraction and Reporting

One researcher (KS) extracted data from the included studies into a pre-specified data-extraction table (DET) in Microsoft Excel. A second researcher (CR) quality-checked 100% of the extracted data against the original publications for accuracy. For consistency, where available, data were extracted for 12 months or for the latest timepoint available. A list of data points extracted is provided in **Supplementary Methods 2**. For studies reporting mean adherence, the population-weighted mean across studies was calculated. The population weights were the sample size divided by the total number of patients with mean adherence over 12 months in the included studies. The weights were multiplied by the adherence in each study, then summed to obtain the weighted mean.

### Risk of Bias

As part of the data-extraction process, one researcher (KS) quality-assessed the RCTs for bias using the Cochrane Risk of Bias tool 2 (RoB2). Observational studies, including cohort studies, surveys, and cross-sectional studies, were quality assessed using the Newcastle–Ottawa Scale (NOS) for observational studies. A second researcher (CR) checked all of the quality assessment ratings; any differences were resolved through discussion. Risk-of-bias assessment was not conducted for studies identified in the targeted search of the Chinese literature.

## Results

### Study Selection

**Figure 1:**
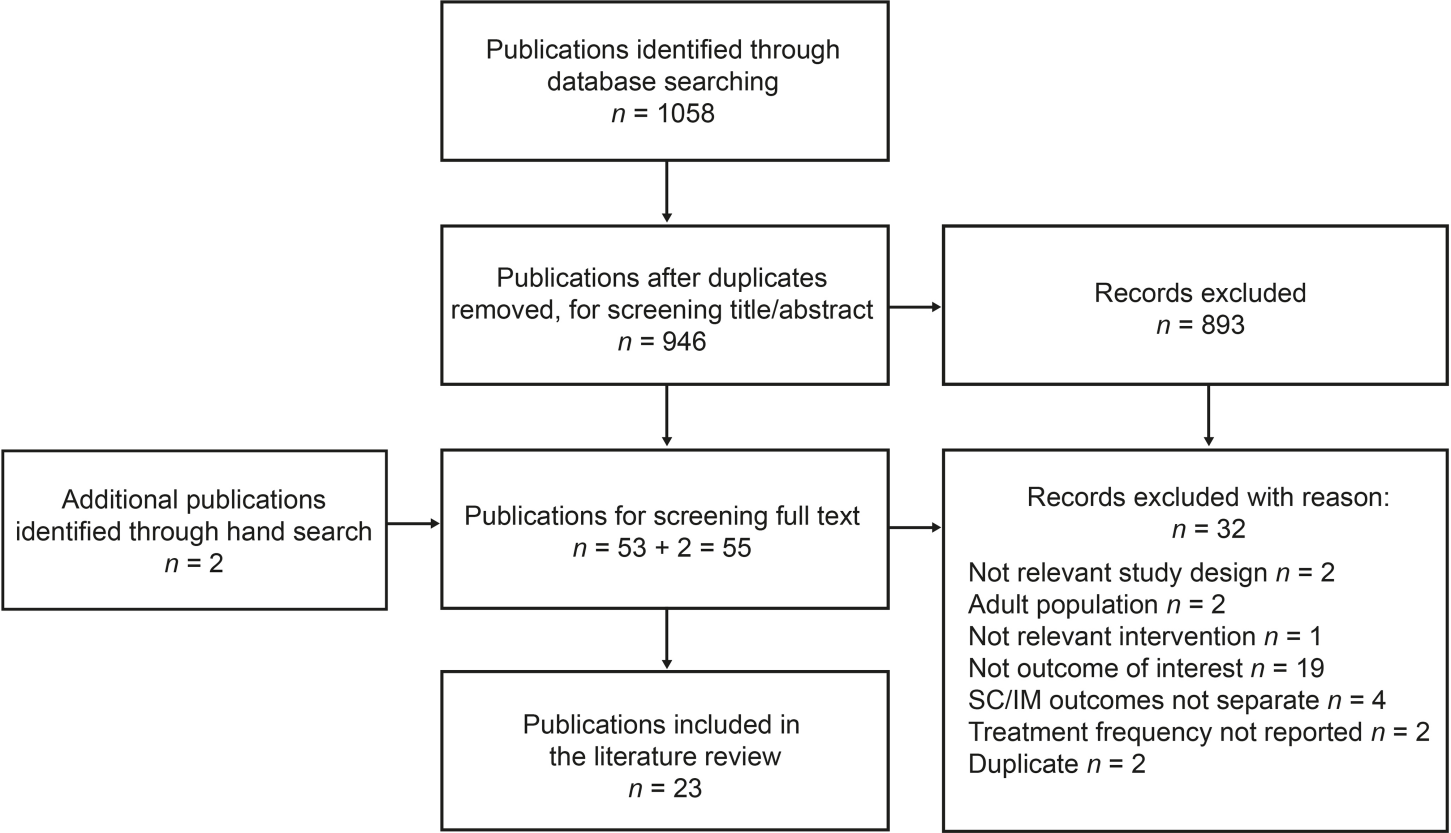
PRISMA diagram of the literature-screening process. PRISMA, Preferred Reporting Items for Systematic Reviews and Meta-Analyses.

A total of 1058 references were identified from the database searches; following removal of duplicates, 946 references underwent title/abstract screening, and 893 were excluded (**Figure 1**). Full-text screening was then performed on 55 references (53 from literature databases and two identified from hand search), and a total of 23 publications met the inclusion/exclusion criteria and were subjected to data extraction and quality assessment.

### Study Characteristics

The details of each study are provided in **Supplementary Table S3**. Of the 23 studies that met the inclusion criteria, only one study (11) was in an indication (MS) outside of pediatric growth disorders. Study sample sizes ranged from 30 (12) to 13,553 (13) patients, with a median sample size of 95 patients. More than half of the sample population was male in all studies (median 41.5% female) except for the MS study, which was 70% female (11). The mean age of patients among the 17 rhGH studies that reported age ranged from 6 years (for patients receiving daily rhGH) (14) to 12.3 years (15). A total of 6 (26%) studies were from Italy (11, 16–20), five (22%) were from multiple countries (13, 14, 21–23), and three (13%) were from Spain (4, 12, 24). The remaining nine studies were each from a different country (15, 25–32).

Two of the studies were clinical trials. REAL 3 (14) was a Phase 2 randomized, active-controlled, open-label study of short-acting (daily) *vs*. long-acting (weekly, three different dosages) rhGH. SYNERGY (27) was a randomized, open-label study of growth outcomes following 12 months of daily rhGH treatment compared with 6 months’ delay followed by 6 months of daily rhGH; adherence for the 12-month treatment arm is reported in this review. Other than the REAL 3 trial (14), which reported adherence to short-acting *vs*. long-acting rhGH, the remaining 21 rhGH adherence studies were of daily GH.

### Critical Appraisal

The 23 included studies were subject to critical appraisal/quality assessment using the quality assessment tool appropriate for each study type. The reviewers (KS and CR) initially agreed on all ratings except for 4 of the observational cohort studies, which they then discussed until agreement was reached. Both of the RCTs (14, 27) were identified as having a moderate risk of bias using the RoB2 tool (**Supplementary Table S4**). Both RCTs collected self-reported adherence, which could have been subject to bias. Further, the 2018 study by Chung et al. (27) was an open-label study, and the 2020 study by Sävendahl et al. (14) was partially blinded, which could have introduced bias. Study quality for the 18 observational cohort studies was assessed using the NOS (**Supplementary Table S5**). Four studies were rated as good quality, and 14 as fair quality; none of the observational cohort studies had a comparator intervention or control group. Of the 18 studies, 12 did not control for differences among patients in the cohort, which may have influenced results. The quality of the three survey studies (15, 16, 21) was assessed using a modified NOS (**Supplementary Table S6**). One study was of fair quality, and two were of low quality; none of the survey studies reported participation rates or information on non-respondents.

### Adherence (Definition and Reporting)

Broadly, adherence in these studies was assessed by several different methods (depending on the study): number of administrations/prescribed doses, as monitored by a medical device [*n* = 12/23 (52.2%)], self-reported number of missed doses for a given time period [*n* = 7/23 (30.4%)], or quantity of pharmacy fills or product supplied/quantity prescribed (*n* = 4/23 [17.4%]). Details of the definition of adherence used in each study are provided in **Supplementary Table S7**. All of the rhGH studies that monitored adherence using a medical device used the easypod™ (Merck) electronic drug-delivery device. The studies also differed in terms of how they reported adherence; some studies reported mean or median adherence rate, whereas others reported the percentage of patients within different pre-specified categories (which varied across studies). Some studies reported adherence using more than one of these measures.

### Mean Adherence

**Figure 2:**
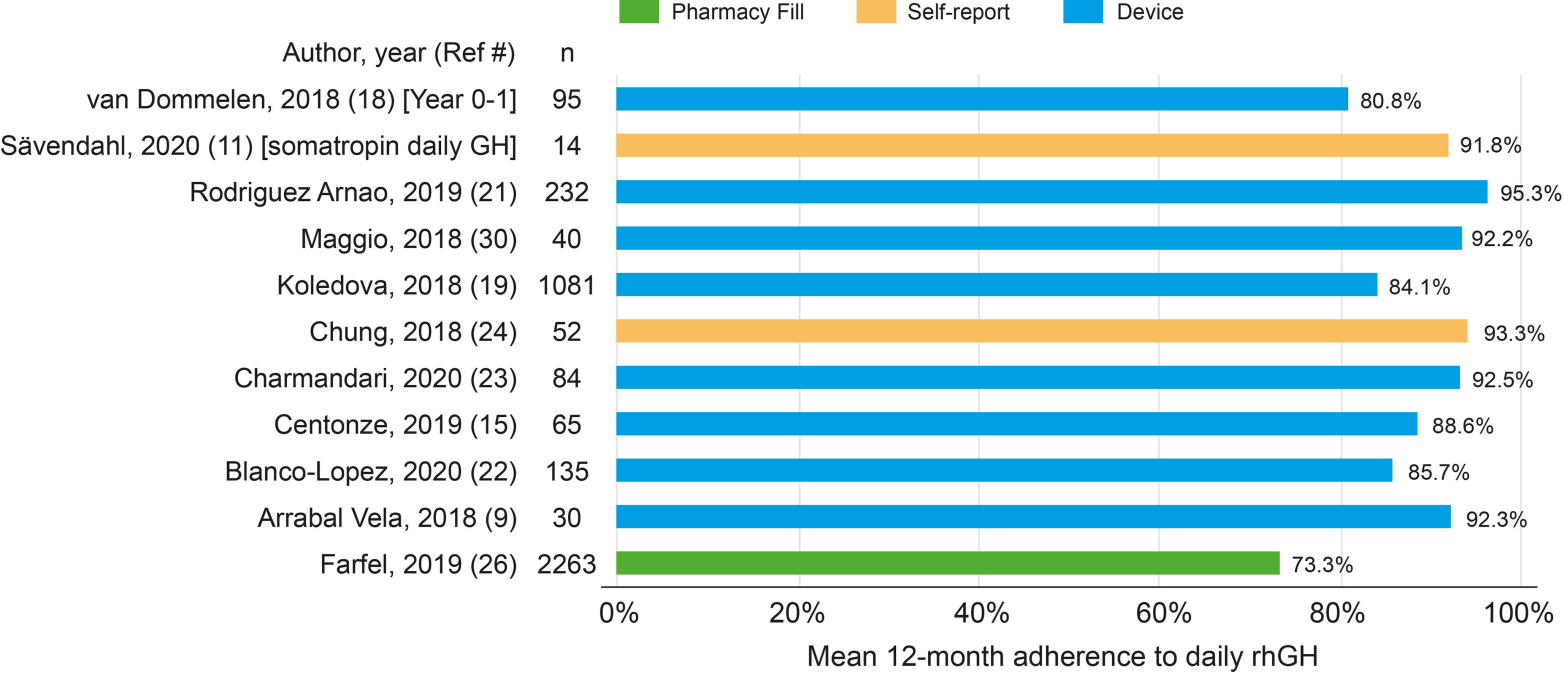
Mean 12-month adherence among 11 rhGH studies. rhGH, recombinant human growth hormone.

Of the 22 rhGH studies, 11 reported the 12-month mean adherence rate to daily rhGH (**Figure 2**). One study compared daily rhGH against weekly rhGH (three different doses) and found that the mean 12-month adherence rate was 91.8% for daily treatment *vs*. 97.5%, 98.6%, and 96.3% for the weekly doses (14); the remaining rhGH publications reported adherence to daily rhGH. The population-weighted mean adherence rate among the 11 studies was 79.3%. Mean adherence rates across the 11 studies ranged from 73.3% (29) to 95.3% (24).

Of the 11 studies, Farfel et al. was different from the others because it measured adherence to rhGH based on the number of months with a pharmacy fill (and not days’ supply) (29). Eight of the 11 studies measured adherence using a medical device (all easypod™); the mean adherence rate across these eight studies ranged from 80.8% (in year 0–1, *n* = 95 patients) (22) to 95.3% (*n* = 232 patients) (24). In the single study on MS, the mean 12-month adherence rate (measured using a medical device; **Supplementary Table S7**) to interferon beta-1a among 40 pediatric patients was 67.5% (11).

### Median Adherence

**Figure 3:**
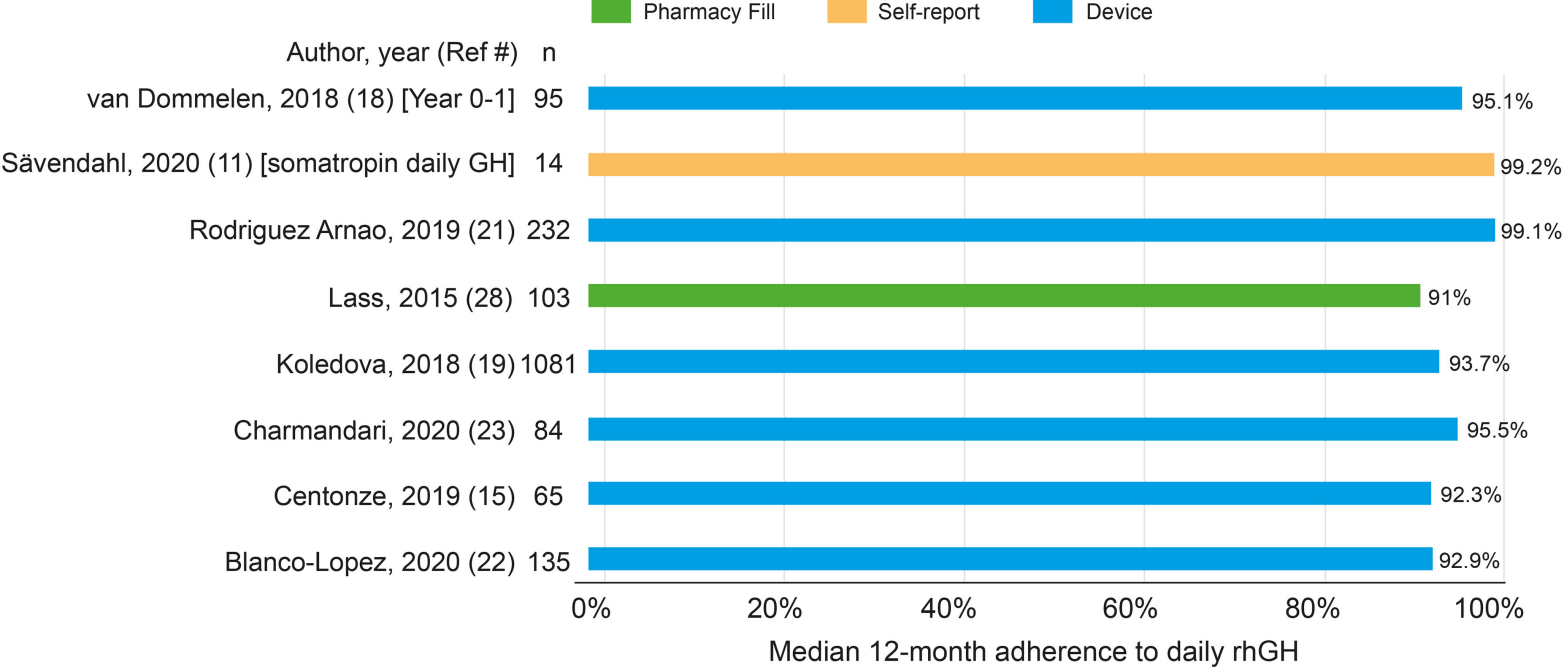
Median 12-month adherence among eight rhGH studies. rhGH, recombinant human growth hormone.

A total of 8/22 rhGH studies reported median adherence to daily rhGH (**Figure 3**), and of these, most (6/8) studies used a medical device (easypod) to monitor adherence. Median adherence to daily rhGH was high, ranging from 91% in a single-center retrospective observational study from Germany (31) to 99.2% in an open-label, multicenter RCT (14) (**Figure 3**).

### Categorical Measures of Adherence

**Figure 4:**
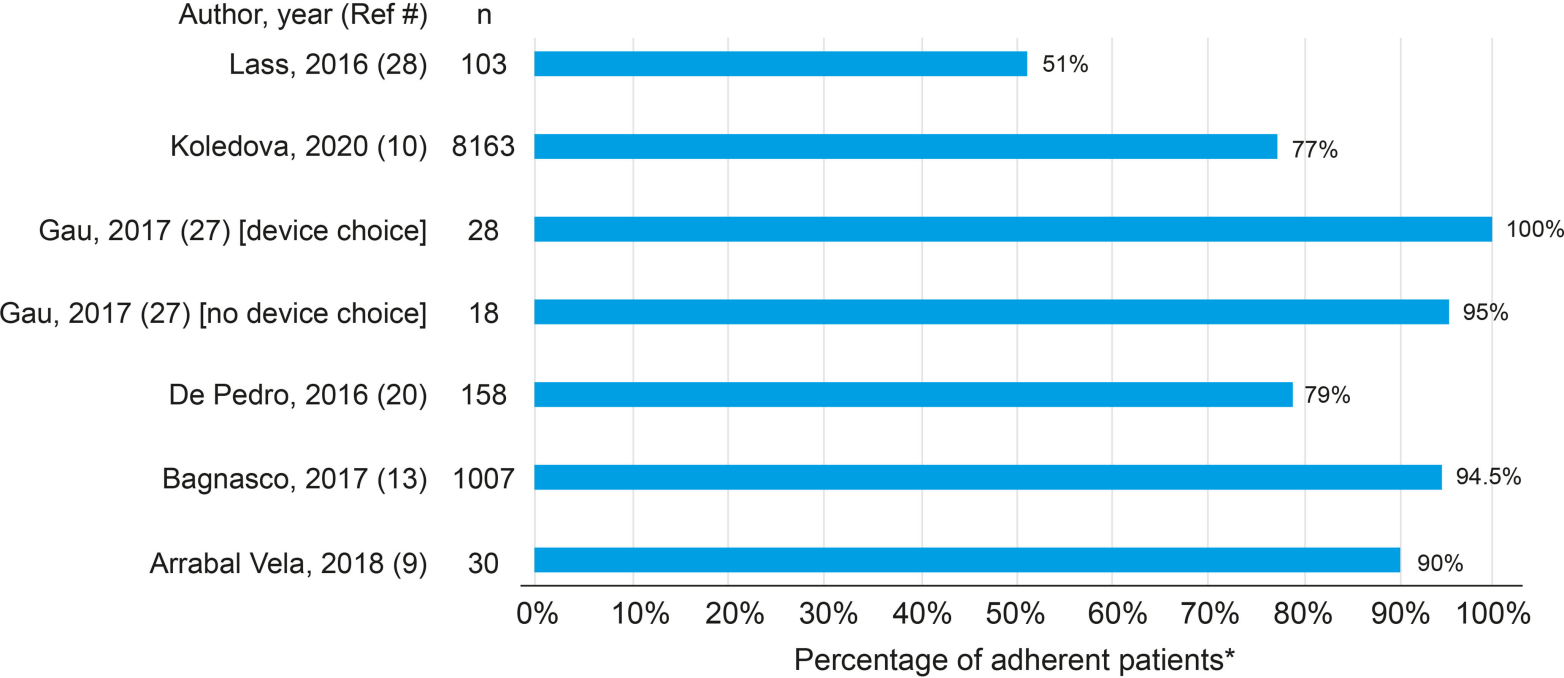
Percentage of patients with adherence > 85%. *Adherence > 85% defined as missed < 1 dose per week, or patients were administered or had in possession > 85% of prescribed doses.

Many of the studies referred to adherence categories, using terms such as “adherent” or “non-adherent” or “poor,” “fair,” “good,” or “excellent” adherence; details are described in **Supplementary Table S7**. Of the studies reporting adherence as a category, the largest number of studies defined patients as adherent (having “good adherence”) to daily treatment if they missed <1 dose per week or were administered >85% of prescribed doses (12, 13, 20, 24, 26, 30, 31). Six studies reported the percentage of patients with adherence >85% or with <1 missed dose per week (**Figure 4**) (4, 12, 13, 16, 30, 31). In a 2015 study of 103 patients that defined adherence as possession of >85% of prescribed doses (pharmacy fills), Lass and Reinehr reported that 51% of patients were considered adherent (31). In the largest study population included in this review, Koledova et al. reported in 2020 that 77% of 8163 patients with 12 months of data had injected >85% of the prescribed dose of rhGH, as measured by the injection device (13).

A clinical trial by Chung et al. (27) defined non-adherence as receipt of <75% of expected injections based on self-reported diary entries. Based on this definition, adherence (receipt of ≥75% of expected injections) in patients who received treatment for 12 months was 93.27%. Using the same definition, adherence in the control group (patients untreated for the first 6 months and then treated with rhGH for 6 months) was 95.69% (27). Adherence would have been lower if the more commonly used definition, receipt of >85% of expected injections, was used to define adherence.

Two studies used ≥80% to define good adherence (15, 32). The study by Michaelidou et al. (32) was a single-center study from the United Kingdom that measured adherence based on the proportion of days covered, defined as the quantity of jet-injector device-heads delivered, multiplied by the length of time each head should last (1 week), divided by the number of days rhGH treatment was prescribed during the treatment period (**Supplementary Table S7**) (32). According to this definition, 57.5% of patients had sufficient device-heads to be considered adherent.

### Easypod Connect Observational Study (ECOS) and Other Easypod Studies

The medical device easypod was the most commonly used method of measuring adherence. Of the 22 included rhGH studies, 11 used easypod to measure adherence to somatropin; these studies (identified by **) are described and adherence reported in **Supplementary Table S7**; most of these studies were sponsored by Merck. ECOS was a Phase 4, open-label study spanning 5 years and 24 countries that measured adherence to rhGH (Saizen, Merck) administered by the easypod. Mean adherence in the primary ECOS (23), which included patients with GHD, small for gestational age, and Turner syndrome from 24 countries, was 84.1%, with a median adherence of 93.7% (23).

In addition to the primary ECOS (23), five different ECOS sub-analyses (all prospective observational studies) met the SLR inclusion criteria. One of these was a 24-country sub-analysis restricted to children with idiopathic isolated GHD (22), wherein the mean (standard deviation [SD]) adherence in the first year was 80.8% (31.1%) with a median of 95.1%. In the second year, the mean (SD) adherence was 81.5% (23.0%), and the median was 92.9%. The remaining four ECOS were specific to certain countries (Mexico, Greece, Spain, and Italy), with a mean adherence ranging from 85.7% (Mexico) (25) to 95.3% (95% confidence interval: 93.3–97.2) (Spain) (24). Median adherence was above 92% for all four studies and ranged from 92.3% (Italy) (18) to 99.1% (Spain) (24).

In addition to the six ECOS, there were five other studies that used easypod to collect adherence data; three from Italy (17, 19, 20), one from Spain (12), and one large multi-country study of 13,553 patients described above (13). All were retrospective studies except for one by Loche et al. (19), which was a prospective observational study. Of the three studies that reported mean adherence rate, values ranged from 70% ± 13% (time period not reported) (17) to 96% over 12 months (20).

### Chinese Literature Survey

The targeted search of the Chinese literature identified two publications, only one of which met the SLR inclusion criteria. A 2020 randomized controlled trial by Li and Liu compared long-acting polyethylene glycol rhGH with short-acting daily rhGH. The investigators reported the number of missed doses out of the total number of prescribed doses for each treatment arm over 26 weeks (33). Adherence was very high in both treatment groups (>99%); however, the difference between adherence in the combined long-acting rhGH (4/1066 missed doses, or 99.62% adherence) and short-acting rhGH (70/7280 missed doses, or 99.04% adherence) was statistically significant (33).

### Barriers to Adherence

Several barriers to adherence were identified in the included publications. Older age/adolescence was identified as a barrier in five studies (4, 12, 20, 31, 32). One study by Maggio et al. that looked at different age groups found a lower adherence in patients aged ≤9 years or ≥14 years compared with those aged 10–13 years (20). A longer treatment duration was also associated with a lower adherence, as described in six studies (4, 12, 16, 20, 29, 31). A number of barriers were identified in single studies, and these included: self-administration (*vs*. caregiver-administered) of rhGH (31); lower education level of the patient’s mother (4); prescribed seven doses of rhGH compared with six doses administered per week (20); and lack of family education/awareness and engagement (16). Device design could potentially also be a barrier as patients who considered the injection device to be ‘not convenient at all’ were less likely to be adherent when compared with patients who considered the device ‘very convenient’ (16). None of these studies reported statistically significant differences in adherence by gender or growth outcomes.

### Recommendations for Improving Adherence

The studies identified in this review recommended multiple methods of improving adherence (**Table 2**). Broadly, most of the recommendations were aimed at interactions between patients and the health care team, in addition to the use of reminder tools to improve adherence. Recommendations were also proposed for the injection device and the drug product (**Table 2**). Recommendations for the injection device focused on improving device design in order to reduce pain and needle anxiety and to increase convenience. It was suggested that adherence might also be improved if the drug product did not require cold storage and was able to be injected less frequently than once per day.

**Table 2:** Recommendations for improving adherence.

Interaction with the healthcare team
Age-appropriate education and awareness of treatment objectives and the importance of adherence, particularly when administration shifts from caregiver to child (16, 29, 31)
Increased patient engagement, such as in selecting the device (16, 30) and in shared decision-making (11)
Ongoing feedback of treatment efficacy, to encourage compliance (16)
**Adherence reminder tools**
Electronic injection reminders, mobile phone reminders, and applications (13)
Gamified interventions that include goal setting, incentive-based engagement, and education (13)
Real-time monitoring of adherence using internet-connected devices (13)
Use of an electronic monitoring device to track adherence (12, 13, 23)
**Device**
Improvements in device design that reduce pain (29)/needle anxiety (32) and increase convenience (16)
**Product**
A product that does not require cold storage (i.e., storage-flexible rhGH) (21)
Reduction in the number of injections from daily to weekly (14)

rhGH, recombinant human growth hormone.

## Discussion

This SLR was undertaken to provide an overview of pediatric patient adherence to rhGH and other injectable treatments as well as to identify some of the factors currently affecting adherence. Since 2010, there have been two SLRs (1, 8) reviewing non-adherence, the most recent of which was published in 2018. We initiated this SLR to capture the most recent data on adherence in the literature. The studies that met the inclusion criteria for this SLR included those with small (30 patients) and large (up to >13,000 patients) sample sizes, and the mean age of patients in the studies ranged from 6 years to 12.3 years. Although studies in other chronic conditions such as MS, JIA, or IBD were eligible for inclusion in this review, of the studies that met the inclusion criteria, only one study was in an indication (MS) outside of GHD. While this SLR is focused primarily on GHD, it is possible that the motivation for taking an injectable therapy may differ between patients with GHD and those with a chronic inflammatory condition. A key strength of this SLR was the fact that the included studies spanned a large number of different countries in several geographic regions, including the Americas, Europe, Asia, and the Middle East. This SLR is also one of the first to evaluate adherence in patients receiving the next generation of long-acting GH treatments. Based on the included studies, the overall trend observed was that pediatric patients had a high adherence to rhGH therapy. However, this observation should be interpreted in light of the limitations of this SLR.

One of the main limitations of this SLR was the difficulty in comparing studies, due to the different methods used for measuring and defining adherence. Although most studies reported adherence over 12 months, a few studies reported adherence over a shorter time period or failed to report the observation period, further limiting comparability among studies. More than half of the studies did not report mean or median adherence, and for the studies with categorical measures, the category definitions and cut-offs varied based on the study. Another potential limitation is the possible influence of the Hawthorne effect on the study findings; that is, patients knowing they were in a study may have been more likely to engage in desired behavior (adherence) than if they were not monitored. The fact that a risk-of-bias assessment was not performed on the single Chinese publication identified in the targeted search was also a study limitation.

From the 11 studies reporting 12-month mean adherence, adherence ranged from 73.3% (29) to 95.3% (24); the population-weighted mean adherence across the 11 studies was 79.3%. As stated above, of these 11 studies, the 2019 study by Farfel et al. was different from the others because adherence was based on the number of months with a pharmacy fill, rather than days’ supply (29). This method of measurement may have underestimated patients’ access to rhGH in the event that they received doses from sources other than through their pharmacy benefit (e.g., from a physician). This method may have also over-reported adherence if there was an overlap whereby the months’ supply of rhGH exceeded the prescribed amount. Further, although patients may have picked up or received their treatment, it cannot be assumed that all doses were injected.

A total of six rhGH studies reported the percentage of patients who missed <1 dose per week (>85% of prescribed doses injected or in possession); adherence according to this definition ranged from 51% (based on pharmacy fills) (31) to 100% (self-reported) among patients able to select their choice of injection device. The high adherence rate in the two clinical trials, which used diaries to self-report adherence, could have been influenced by a potential Hawthorne effect and may not be reflective of real-world adherence to rhGH. The high adherence associated with the use of injector devices may have also been influenced by patients’ awareness that utilization data were being sent to their health care provider or to researchers. This suggests that the use of injector devices with data reviewed regularly by a health care provider may be an effective method to increase/improve adherence to treatment. Additional studies of adherence among patients using other device types are needed to clarify the influence of the Hawthorne effect and the ramifications for routine clinical practice.

Of interest is the study by Mohseni et al. in 2018, which compared different methods of reporting adherence (15). Based on the self-reported, eight-item Morisky Medication Adherence Scale, 56.7% of children (aged 2–12 years) and 57.9% of adolescents (13–19 years) had moderate-to-high adherence to rhGH (15). Using the “auto-compliance method,” based on self-reported number of injections divided by the number of injections prescribed (with ≥80% of prescribed injections reported as received being considered “adherent”), adherence rates were 95.2% among children and 95.5% among adolescents (15). The large difference in adherence seen in this study illustrates how the choice of methods used to measure and define adherence can affect reported adherence. Similarly, in the two published SLRs on adherence to rhGH, adherence rates also varied widely depending on the measures and definitions used, from 18% to 95% in the 2013 review by Fisher and Acerini (1), and from 29% to 93% in Graham et al. (8).

**Figure 5:**
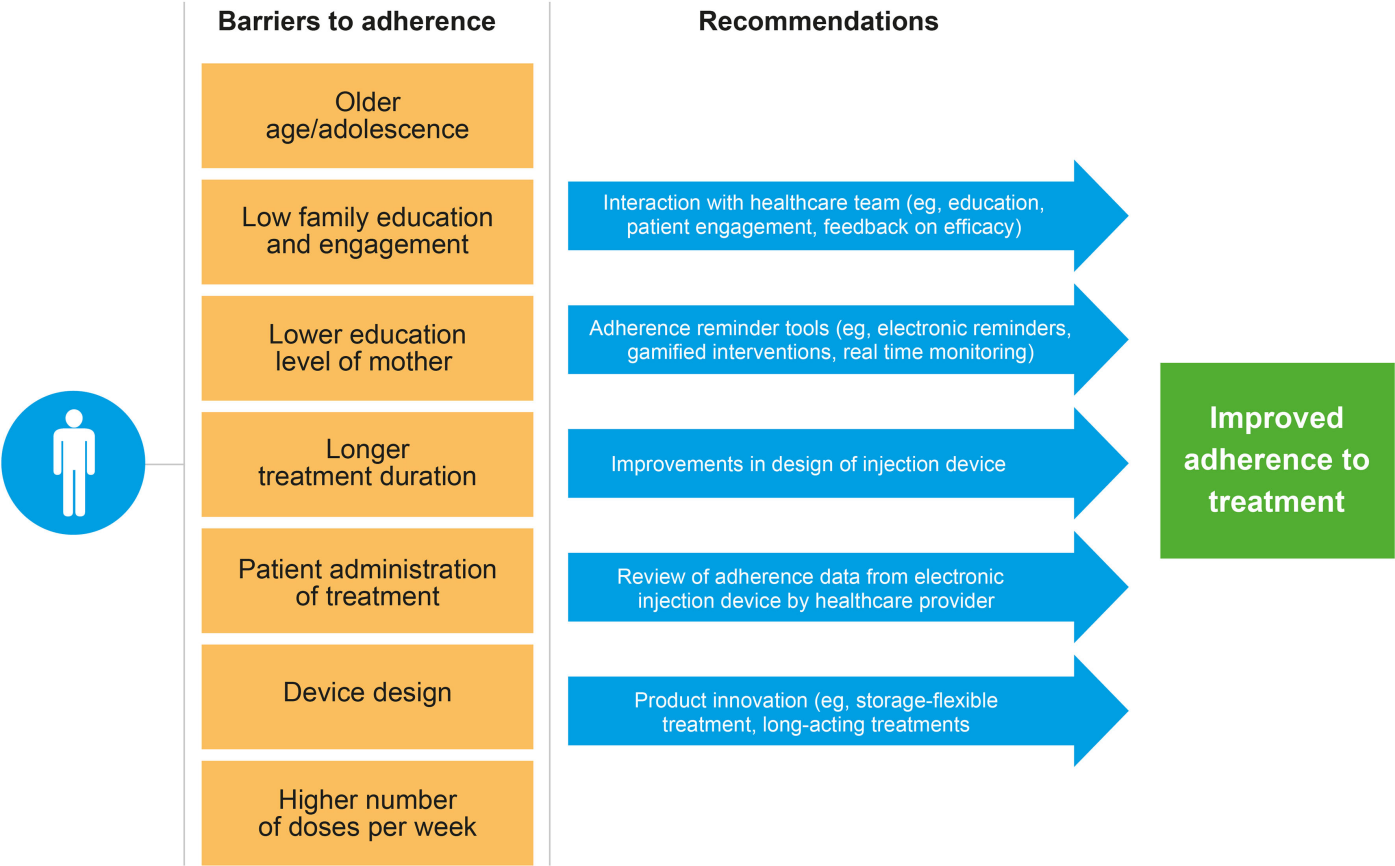
Barriers to adherence and recommendations for improving adherence (identified in the included studies).

The barriers to adherence (**Figure 5**) identified in this review were similar to those reported by Fisher et al. in 2013 (1). The previous SLR identified injection-related factors associated with non-adherence as being perceived difficulty of injections, lack of choice of injection device, short duration of prescriptions, and discomfort (1). Of the 20 modifiable factors identified in Graham et al. (8), those related to poor adherence to rhGH included injection-related pain and discomfort, poor administration technique, forgetting injections, disruption in supply of injections due to short duration of prescriptions, and being away from home (8). Based on our extensive experience in clinical practice, we suggest that dissatisfaction with treatment outcome, a lack of understanding of the consequences of missed doses, and poor knowledge and understanding of the disease condition also constitute substantial barriers to adherence. Non-modifiable risk factors to adherence such as gender, age, race, severity, and duration of disease may also affect the impact of modifiable risk factors and should be carefully considered in studies evaluating adherence.

The studies included in this review recommended multiple methods of improving adherence, focused in particular on device choice, patient and caregiver education and engagement, reminders, and utilization monitoring. The one study that compared adherence to daily *vs*. weekly rhGH also suggested that weekly dosing could improve adherence compared with daily dosing. The increasing emergence of long-acting rhGH treatments has the potential to improve adherence and treatment outcomes. One potential consideration for long-acting rhGH products is that the consequences of missing an injection are more severe, given that missing one injection is equivalent to missing seven daily rhGH injections. A number of study recommendations focused on technological innovations for the injection device, indicating great interest in the potential of technology to improve adherence. In addition to those already mentioned, features such as automatic recording of missed doses and automatic revision of subsequent doses (to compensate for a missed dose) may also help patients to remain adherent. The utilization of growth-prediction models (34) as part of patient consultation may also encourage adherence. Patients who fail to observe an adequate response to treatment may be less likely to adhere to treatment; optimizing treatment response using growth-prediction models may potentially help empower patients and reduce patient dissatisfaction with treatment, thereby encouraging patients to remain adherent. However, the use of prediction models to improve adherence requires further study. Clinicians should consider reassessing a patient’s diagnosis if poor response is observed, particularly if treatment adherence has been high. There are several other interventions that focus more on behavior modification such as adherence therapy, cognitive behavioral therapy, cognitive adaptation training, family interventions, and psychoeducation/monetary-based interventions that have not been explored to date in the field of rhGH therapy. Furthermore, given that the previous studies have already identified the risk factors that predispose patients to impaired adherence, future studies should explore a more individualized approach where interventions could be targeted at those who are at a high risk of poor adherence.

### Study Recommendations Based on SLR Findings

One of the main findings from this review was the substantial heterogeneity in the way adherence was defined, measured, and reported in recent publications on rhGH. This heterogeneity makes it difficult to compare studies and to uncover trends and patterns across the literature. Therefore, we propose the following recommendations to enable more effective characterization/comparison of adherence data across future studies and publications. First, we propose standardizing the way adherence is defined, measured, and reported. In addition, adoption of standardized study designs, including adherence measures, definitions of “adherent” *vs*. “non-adherent,” and outcomes, is also recommended. Quantitative data describing the impact of missed doses on growth would also be valuable for determining the threshold of adherence to be reported. Furthermore, at a minimum, studies should report the presence or absence of any association between adherence and study duration, patient age, and rhGH indication. Lastly, in studies where the primary purpose is to study adherence, adherence should be measured using more than one method — agreement between different methods in the same study should be assessed. We believe that these measures should help address the substantial heterogeneity among the studies identified in the published literature.

## Conclusions

Reported adherence was >80% in many studies, particularly in those that used an injector device or diaries for self-reporting; adherence among patients who are not being actively monitored or who are not participants in a study may be lower, due to the Hawthorne effect. Among the 22 rhGH studies included in this review of the published literature, there was substantial heterogeneity in how adherence to rhGH was defined, measured, and reported. Standardization of how adherence is defined and reported, as well as in study design and outcomes measured, would enable more extensive comparisons to be made among different pediatric populations. Factors that could improve adherence include patient and caregiver education and active involvement in the treatment plan, including choice of device. Once available, long-acting rhGH formulations, which would allow weekly instead of daily dosing, may also result in improved adherence to treatment. It will be important to use proper comparisons of adherence between daily rhGH and long-acting rhGH preparations to demonstrate this relationship.

## Data Availability Statement

The original contributions presented in the study are included in the article/**Supplementary Material**. Further inquiries can be directed to the corresponding author.

## Author Contributions

RG, SFA, MM, DL, TT, and BSM contributed to the design, planning, and conception of the study, as well as data interpretation and manuscript development. DL (fluent in Chinese) conducted the literature search in the Wan Fang database. All authors reviewed manuscript drafts and have reviewed and approved the final version for submission.

## Funding

This study was funded by Pfizer.

## Conflict of Interest

RG: employee of and owns shares/options in Pfizer. MM: research support from Pfizer and Merck Serono and consultant for Pfizer, Novo Nordisk, Merck Serono, Ferring, Biomarin, and Ascendis. SFA: unrestricted research and education support from Diurnal, Neurocrine Biosciences, Novo Nordisk; chief investigator for Acerus; and consultant for Sanofi. TT: consultant for JCR pharmaceuticals. BSM: consultant for AbbVie, Ascendis Pharma, BioMarin, Merck Serono, Novo Nordisk, Orchard Therapeutics, Pfizer, Sandoz, Tolmar and Vertice Pharma and research support from Alexion, Abbvie, Amgen, Lumos Pharma, Novo Nordisk, OPKO, and Pfizer.

The authors declare that this study received funding from Pfizer. The funder had the following involvement in the study: input into study design, interpretation of data, and preparation of the manuscript. The authors had final authority on all aspects of the manuscript content and development, including on the choice of journal.

The reviewer CG declared a past co-authorship with several of the authors (MM and SFA) to the handling editor.

## Publisher’s Note

All claims expressed in this article are solely those of the authors and do not necessarily represent those of their affiliated organizations, or those of the publisher, the editors and the reviewers. Any product that may be evaluated in this article, or claim that may be made by its manufacturer, is not guaranteed or endorsed by the publisher.

## Abbreviations

ECOS, easypod™ connect observational study; GH, growth hormone; GHD, growth hormone deficiency; hGH, human growth hormone; IBD, inflammatory bowel disease; IGF-1, insulin-like growth factor-1; ISS, idiopathic short stature; JIA, juvenile idiopathic arthritis; MS, multiple sclerosis; NOS, Newcastle–Ottawa Scale; RCT, randomized controlled trials; rhGH, recombinant human growth hormone; SC, subcutaneous; SD, standard deviation; SDS, standard deviation scores; SLR, systematic literature review.
