# Supplementary material for: Treatment Adherence to Injectable Treatments in Pediatric Growth Hormone Deficiency Compared With Injectable Treatments in Other Chronic Pediatric Conditions: A Systematic Literature Review
Source: Front Endocrinol (Lausanne). 2022 Mar 1;13:795224. doi: 10.3389/fendo.2022.795224 (PMC8921265; doi:10.3389/fendo.2022.795224)
Supplement: Supplementary file 1 [file DataSheet_1.docx]

**Supplementary Materials**

**Supplementary Methods 1: Search Strategy**

Searches were run on October 6, 2020 in Embase 1974 week 1 to 2020 week 40, using the search strategy below, in the Ovid interface (Table S1).

**Table S1** | Embase search strategy.

| **Step** | **Search string** | **Hits** |
| --- | --- | --- |
| 1 | (child* or p?diatric or infant or juvenile or youth or prepubertal or pre-pubertal or preadolescen* or pre-adolescen* or preteen* or pre-teen* or preschool* or  pre-school* or adolescen* or pubertal or teen*).mp. | 3,868,709 |
| 2 | (growth hormone or growth-hormone or human growth hormone or somatotro?in* or somatotro?ic or growth hormone deficiency or growth hormone disorder or growth hormone disease or growth hormone condition or growth hormone insufficiency or achondroplasia or Noonan syndrome or SHOX deficiency or short stature homeobox-containing gene deficiency or small for gestational age or idiopathic short stature or Turner syndrome or Prader?Willi  syndrome or chronic renal insufficiency).mp. | 132,549 |
| 3 | (growth hormone or growth-hormone or rGH or rhGH or human growth hormone or recombinant growth hormone or somatropin or Genotropin or Saizen or Zomacton or NutropinAq or Nutropin or Norditropin or Serostim or  Omnitrope or Humatrope).mp | 97,396 |
| 4 | 2 and 3 | 96,842 |
| 5 | exp Arthritis, Juvenile/ | 21,442 |
| 6 | ((Juvenile adj2 arthritis) or (still* adj2 disease) or JIA or JRA or sJIA or soJIA).mp | 37,092 |
| 7 | exp inflammatory bowel disease/ | 153,190 |
| 8 | inflammatory bowel disease.mp | 85,885 |
| 9 | or/5-8 | 209,318 |
| 10 | (etanercept or Enbrel or adalimumab or Humira or Certolizumab pegol or Cimzia or Infliximab or remicade or rituximab or Rituxan or Golimumab or Simponi or canakinumab or abatacept or Orencia or anakinra or Kineret or tocilizumab or  Actemra).mp | 168,161 |
| 11 | (anti-TNF* or TNFi* or (TNF* adj blocker) or (TNF* adj inhibit*)).ti,ab | 33,280 |
| 12 | (DMARD* or disease modifying).ti,ab. | 39,613 |
| 13 | or/10-12 | 212,597 |
| 14 | 9 and 13 | 36,055 |
| 15 | exp Multiple Sclerosis/ | 130,242 |
| 16 | multiple sclerosis.mp | 142,873 |
| 17 | (interferon or glatiramer acetate or Copaxone or Glatopa or Avonex or Rebif or  Betaseron or Extavia or peginterferon or Plegridy).mp. | 402,353 |
| 18 | (DMARD* or disease modifying).ti,ab | 39,613 |
| 19 | 15 or 16 | 142,873 |
| 20 | 17 or 18 | 438,209 |
| 21 | 19 and 20 | 27,608 |
| 22 | (adheren* or nonadheren* or non-adheren* or non adheren* or complian* or noncomplian* or non-complian* or non complian* or persistence or nonpersisten* or non-persisten* or non persisten* or concordan* or discordan*  or continu* or discontinu* or dis-continu* or cessation).mp. | 2,462,209 |
| 23 | 1 and 4 and 22 | 2911 |
| 24 | 1 and 14 and 22 | 2627 |
| 25 | 1 and 21 and 22 | 305 |
| 26 | 23 or 24 or 25 | 5806 |
| 27 | ((animal$ not human$) or in vitro or nonhuman).sh,hw. | 8,496,500 |
| 28 | (animal* or in vitro or tissue* or murine or mouse or mice or swine* or pig* or porcine or rat or rats or rodent* or monkey or simian or ape or dog or canine* or cats or cat or feline* or cow or bovine or horse or equine or fish or piscine or  rabbit*).ti | 2,927,994 |
| 29 | (conference abstract or congress or editorial or comment* or letter or note).pt.  or (congress or conference or symposium).ti,sh | 6,582,914 |
| 30 | (cancer* or chemo* or radiotherapy* or radiation or carcinoma* or sarcoma*or  tumor* or tumour*).ti,ab. | 3,965,213 |
| 31 | or/27-30 | 16,976,127 |
| 32 | 26 not 31 | 2852 |
| 33 | limit 32 to yr="2015 -Current" | 780 |
| 34 | Remove duplicates from 33 | 763 |

Searches were run in Ovid MEDLINE® and Epub Ahead of Print, In-Process & Other Non-Indexed Citations, Daily and Versions® 1946 to October 1, 2020, using the search strategy below. Searches were run October 6, 2020.

Searches were run in Ovid MEDLINE® and Epub Ahead of Print, In-Process & Other Non-Indexed Citations, Daily and Versions® 1946 to October 01, 2020, using the search strategy below (Table S2). Searches were run 6 October 2020.

**Table S2** | Medline search strategy.

| **Step** | **Search string** | **Hits** |
| --- | --- | --- |
| 1 | (child* or p?diatric or infant or juvenile or youth or prepubertal or pre-pubertal or preadolescen* or pre-adolescen* or preteen* or pre-teen* or preschool* or  pre-school* or adolescen* or pubertal or teen*).mp. | 4,219,342 |
| 2 | (growth hormone or growth-hormone or human growth hormone or somatotro?in* or somatotro?ic or growth hormone deficiency or growth hormone disorder or growth hormone disease or growth hormone condition or growth hormone insufficiency or achondroplasia or Noonan syndrome or SHOX deficiency or short stature homeobox-containing gene deficiency or small for gestational age or idiopathic short stature or Turner syndrome or Prader?Willi syndrome or chronic renal insufficiency).mp. | 107,120 |
| 3 | (growth hormone or growth-hormone or rGH or rhGH or human growth hormone or recombinant growth hormone or somatropin or Genotropin or Saizen or Zomacton or NutropinAq or Nutropin or Norditropin or Serostim or Omnitrope or Humatrope).mp | 75,842 |
| 4 | 2 and 3 | 75,537 |
| 5 | exp Arthritis, Juvenile/ | 10,464 |
| 6 | ((Juvenile adj2 arthritis) or (still* adj2 disease) or JIA or JRA or sJIA or soJIA).mp | 23,431 |
| 7 | exp Inflammatory Bowel Diseases/ | 81,488 |
| 8 | inflammatory bowel disease.mp | 47,762 |
| 9 | or/5-8 | 124,900 |
| 10 | (etanercept or Enbrel or adalimumab or Humira or Certolizumab pegol or Cimzia or Infliximab or remicade or rituximab or Rituxan or Golimumab or Simponi or canakinumab or abatacept or Orencia or anakinra or Kineret or tocilizumab or Actemra).mp | 58,875 |
| 11 | (anti-TNF* or TNFi* or (TNF* adj blocker) or (TNF* adj inhibit*)).ti,ab | 15,830 |
| 12 | (DMARD* or disease modifying).ti,ab. | 20,371 |
| 13 | or/10-12 | 84,922 |
| 14 | 9 and 13 | 10,619 |
| 15 | exp Multiple Sclerosis/ | 59,304 |
| 16 | multiple sclerosis.mp | 88,777 |
| 17 | (interferon or glatiramer acetate or Copaxone or Glatopa or Avonex or Rebif or  Betaseron or Extavia or peginterferon or Plegridy).mp. | 202,452 |
| 18 | (DMARD* or disease modifying).ti,ab | 20,371 |
| 19 | 15 or 16 | 88,777 |
| 20 | 17 or 18 | 221,609 |
| 21 | 19 and 20 | 10,696 |
| 22 | (adheren* or nonadheren* or non-adheren* or non adheren* or complian* or noncomplian* or non-complian* or non complian* or persistence or nonpersisten* or non-persisten* or non persisten* or concordan* or discordan*  or continu* or discontinu* or dis-continu* or cessation).mp. | 1,848,245 |
| 23 | 1 and 4 and 22 | 1910 |
| 24 | 1 and 14 and 22 | 759 |
| 25 | 1 and 21 and 22 | 204 |
| 26 | 23 or 24 or 25 | 2872 |
| 27 | ((animals not (humans and animals)) or in vitro or nonhuman).sh | 4,709,572 |
| 28 | (animal* or in vitro or tissue* or murine or mouse or mice or swine* or pig* or porcine or rat or rats or rodent* or monkey or simian or ape or dog or canine* or cats or cat or feline* or cow or bovine or horse or equine or fish or piscine or rabbit*).ti | 2,818,702 |
| 29 | (editorial or comment* or letter or note).pt. | 1,917,987 |
| 30 | (cancer* or chemo* or radiotherapy* or radiation or carcinoma* or sarcoma*or tumor* or tumour*).ti,ab. | 3,100,656 |
| 31 | or/27-30 | 10,128,377 |
| 32 | 26 not 31 | 2531 |
| 33 | limit 32 to yr="2015 -Current" | 674 |
| 34 | Remove duplicates from 33 | 619 |

**Supplementary Methods 2: Data-Extraction Elements**

- The following data points were extracted, where available, from eligible studies:
  - Bibliographic details (authors, title, reference number)
  - Study name/registry/database
  - Study design
  - Aim
  - Country
  - Patient eligibility criteria (inclusion/exclusion)
  - Sample size
  - Duration of follow-up
  - Reporting timepoint (timepoint for which data will be extracted)
  - Patient characteristics at baseline
  - Age, gender, ethnicity
  - Clinical indication (GH-related indication, JIA, IBD, MS)
    - For GH-related indications, subgroups may include: GHD, Turner syndrome, SHOX deficiency, small for gestational age (SGA), idiopathic short stature (ISS), Prader–Willi syndrome, neurosecretory dysfunction, intrauterine growth retardation, bioinactive GH, or chronic renal failure
- Intervention/study arm characteristics
  - Treatment, administration route, and scheduling
  - Median/mean duration of treatment
  - Duration of follow-up
- Outcomes:
  - Method of assessing adherence
  - Definition of adherence/non-adherence
  - Adherence prevalence (*n*, %)
  - Prevalence of adherence/non-adherence (*n*, %)
  - Stated barriers to adherence
  - Factors associated with adherence to prescribed treatment
  - Factors not associated with adherence to prescribed treatment
  - Recommendations for improving adherence
- For the Chinese publications identified in the targeted search, data were extracted into a brief modified DET to report key information regarding study population and adherence. One researcher (Dejun Li) extracted the data, translated into English. A second researcher (Shuang Li), fluent in Chinese, quality-checked the extracted data against the Chinese-language publications. Any disagreements were resolved by discussion.

*Refer to abbreviation list on last page*

**Table S3** | Characteristics of included studies (*n*=23).

| **Author, year (sponsor)** | **Country, study name, or data source** | **Study design** | **Study period** | **Population and sample size, *n* (%)** | **% Female** | **Mean age (SD), years** | |
| --- | --- | --- | --- | --- | --- | --- | --- |
| **Interventional studies (*n* = 2)** | | | | | | |  |
| Chung, 2018 (1) (Merck) | Korea, SYNERGY | Randomized multicenter, open-label study | Dec 2012– Mar 2015 | ISS: 89 (100)  Tx: 59 (66.3)  *Control: 30 (33.7)  With 12 mo of data:  ISS: 79 (100)  Tx: 52 (65.8)  *Control: 27 (34.2) | Tx: 49%  *Control: 43% | Tx: 6.79 (1.54)  *Control: 6.83 (1.61) | |
| Sävendahl, 2020 (2) (Novo Nordisk) | Multinational (11 countries), REAL 3 | Phase 2, open-label, multicenter RCT (SOMA dosing was double-blinded; SOMA vs NORD was not blinded) | Mar 2016–Aug 2018 | GHD: 59 (100)  57 full analysis set (with baseline data)  SOMA (0.04 mg/kg/wk): 14  SOMA (0.08 mg/kg/wk):15 SOMA (0.16 mg/kg/wk): 14 NORD Daily (0.034 mg/kg/d): 14 | SOMA (0.04 mg/kg/wk): 50%  SOMA (0.08 mg/kg/wk): 33.3%  SOMA (0.16 mg/kg/wk): 42.9%  NORD daily (0.034 mg/kg/d): 35.7% | SOMA (0.04 mg/kg/wk): 5.8 (1.8)  SOMA (0.08 mg/kg/wk): 5.9 (1.8)  SOMA (0.16 mg/kg/wk): 6.1 (2.3)  NORD Daily (0.034 mg/kg/d): 6.0 (2.0) | |

| **Observational studies (*n* = 21; 20 rhGH studies; 1 MS study)** | | | | | | |  |
| --- | --- | --- | --- | --- | --- | --- | --- |
| Arrabal Vela, 2018** (3) (NR) | Spain, Pediatric Endocrinology Clinic, Hospital General Universitario de Ciudad Real (medical records) | Retrospective, single center | May 2015– May 2016 | Total: 30  GHD: 14 (46.7)  SGA: 14 (46.7)  Other: 2 (6.6) | 33% | 6.09 (range 4.92–7.25) | |
| Bagnasco, 2017 (4) (Novo Nordisk) | Italy, SIEDP/ISPED (46 centers, medical records) | Cross-sectional survey, multicenter | Nov 2015–  May 2016 | Total: 1007  Parents: 771 (76.6%)  Patients: 221 (21.9%)  Unknown: 15 (1.5%)  Indication: NR (rhGH recipients) | NR | Mode: 14–15 (range 6–15) | |
| Blanco-Lopez, 2020** (5) (Merck) | Mexico, ECOS (sub-analysis) | Prospective, multicenter | Nov 2010–  Feb 2016 | Total: 147  GHD: 118 (80.3)  SGA: 24 (16.3)  TS: 5 (3.4) | 43.2% | 9.96 (3.41) | |
| Cardinale, 2019** (6) (Merck) | Italy (6 centers, medical records) | Retrospective, multicenter | Jan 2015–  Sep 2015 | Total: 90  GHD: 83 (92)  SGA: 5 (6)  TS: 2 (2) | 42% | 11.9 (3.04) | |
| Centonze, 2019** (7) (Merck) | Italy, ECOS (sub-analysis) | Prospective, multicenter | NR | GHD: 73 (100) | 47.9% | 9.78 (3.20) | |
| Charmandari, 2020** (8) (Merck) | Greece, ECOS (sub-analysis) | Prospective, multicenter | NR | Total: 88  GHD: 78 (88.6)  SGA: 4 (4.6)  TS: 3 (3.4)  Other/missing: 3 (3.4) | 37.5% | 10.23 (2.79) | |
| De Pedro, 2016 (9) (Pfizer) | Spain, Endocrinology  Pediatrics, Germans Trias i Pujol University Hospital Badalona (medical records) | Retrospective, single center | 2012 (months NR) | Total: 158  GHD: 121 (76.5)  SGA 37 (23.5) | 36% | 10.6 (2.7) | |
| Dumitrescu, 2020 (10) (Ipsen) | Romania, COMPLIA (13 centers, medical records) | Prospective, multicenter | Jun 2010–Jun 2014 | GHD: 187 (100) | 36.9% | 9.8 (3.6) | |
| Farfel, 2019 (11) (Pfizer) | Israel, Clalit Health Maintenance Organization (claims data) | Retrospective, multicenter | Jan 2006–Dec 2015 | Total: 2263 GHD, SGA, or TS (cannot differentiate): 1612 (71.2)  ISS: 575 (25.6)  CRF: 76 (3.4) | 41% | 8.3 (3.6) | |
| Gau, 2017 (12) (none) | Japan, Kawaguchi Municipal Medical Center (medical records) | Retrospective, single center | No PC: 2008–2010  PC: 2011–2014 | GHD: 46 (100) | 48% | 7.70 (3.12) | |
| Kappelgaard, 2015 (13) (Novo Nordisk) | France, Germany, Italy, USA (surveys) | Cross-sectional survey, multicenter | Apr 2014–Jun 2014 | Storage-flex:  Total: 50^†^ GHD/MPHD: 38  (76)  SGA: 8 (16)  TS: 4 (8)  Other: 1 (2)  Refrigeration-only: Total: 96^†^ GHD/MPHD: 65  (68)  SGA: 21 (22)  TS: 12 (13)  ISS: 3 (3)  RSS: 4 (4)  Other: 3 (3) | Storage-flex:  38%  Refrigeration-only: 40% | Storage-flex:  Mode: 14–17  (range <5–25)  Refrigeration-only:  Mode: 5–7  (range <5–25) | |
| Koledova, 2018** (14) (Merck) | 24 countries, ECOS | Prospective, multicenter | Nov 2010–  Feb 2016 | Total: 1203  GHD: 897 (74.6)  SGA: 207 (17.2)  TS: 82 (6.8)  Other: 17 (1.4) | 41.8% | Median: 10 (range: 1–19) | |
| Koledova, 2020** (15) (Merck) | Multicounty (NR) | Retrospective, multicenter | Jan 2007–  Feb 2019 | Total: 13,553 Indication NR, all patients received rhGH | 42.5% | Boys, by adherence:  ≥85%: 12.8 (5.3)  >56 to <85%: 15.0 (6.6)  ≤56%: 15.3 (7.6)  Girls, by adherence:  ≥85%: 12.2 (5.2)  >56 to <85%: 14.3 (7.9)  ≤56%: 15.8 (10.6) | |
| Lass, 2015** (16) (Merck) | Germany, Vestische Hospital for Children and Adolescents (medical records) | Retrospective, single center | Sep 2014–  Feb 2015 | Total: 103  GHD: 74 (71.8)  SGA: 21 (20.4)  TS: 4 (3.9)  SHOX deficiency: 3 (2.9)  PWS: 1 (1.0) | 35% | At Tx start: 6.6 (2.7)  At study start: 10.1 (range: 8.1–12.2)^§^ | |
| Loche, 2016** (17) (Merck) | Italy, 10 clinical sites (medical records) | Prospective, multicenter | Mar 2010–  Jan 2013 | GHD: 79 (100) | 34% | Median (IQR): 10 (9–12) | |
| Maggio, 2018** (18) (None) | Italy, Paediatric Clinic, G. Di Cristina Children’s Hospital, ARNAS, Palermo (medical records) | Retrospective, single center | 2009–2016  (months NR) | Total: 40  GHD: 26 (65)  SGA: 9 (22.5)  TS: 5 (12.5) | 32.5% | At Tx start: 8.65 (2.81)  At study start: 11.2 (2.3) | |
| Michaelidou, 2019 (19) (Ferring) | UK, University College London Hospitals (pharmacy data) | Retrospective, single center | Jan 2010–Dec 2015 | Total: 52  GHD: 34 (65.4)  TS: 5 (9.2)  Other: 13 (25.0) | 43.3% | 8.50 (3.78) | |
| Mohseni, 2018 (20) (Tehran University of Medical Sciences) | Iran, Tehran University of Medical Sciences (pharmacy data and survey) | Cross-sectional survey, single center | NR | Total: 169 Indication NR, all patients received rhGH | 56.8% | 12.29 (3.09) | |
| Rodriguez Arnao, 2019** (21) (Merck) | Spain, ECOS | Prospective, multicenter | NR | Total: 238  GHD: 144 (60.5)  SGA: 86 (36.1)  TS: 8 (3.4) | 48.3% | At Tx start: 7.9 (3.2)  At study start: 9.0 (3.3) | |
| van Dommelen, 2018** (22) (Merck) | 24 countries, ECOS | Prospective and retrospective, multicenter | NR | GHD (Tx-naïve): 95  (100) | 24% | At study start: 6.3 (2.1) | |
| **Observational studies, agents other than rhGH** | | | | | | |  |
| Ghezzi, 2017 (23) (Merck) | Italy, FUTURE | Prospective | Jan 2012–  Oct 2014 | MS: 40 (100) | 70% | 15 (2.1) | |

*Control consisted of 6 months without rhGH treatment, followed by 6 months with rhGH treatment.

**easypod™ study (includes prospective ECOS and retrospective studies).

†Respondents listed any diagnosis for which they received rhGH; sums exceed 100%.

^§^Mean (range) assumed based on reported values in Lass, 2015 (see Table 1 and text), but not specified in publication.

*Refer to abbreviation list on last page..*

**Table S4** | Quality assessment of RCTs using the Cochrane RoB2.

| **Author, year**  **Trial name Study design** | **Was the allocation sequence random?** | **Was the allocation sequence concealed until participants were enrolled**  **and assigned to interventions?** | **Was knowledge of the allocated interventions adequately concealed from participants and personnel?** | **Was knowledge of the allocated interventions adequately prevented from outcome assessors?** | **Were incomplete outcome data adequately addressed?** | **Are reports of the study free of suggestion of selective outcome reporting?** | **Was the study apparently free of other problems that could put it at a high risk of bias?** |
| --- | --- | --- | --- | --- | --- | --- | --- |
| **Chung 2018 (1)**  SYNERGY  Open-label, randomized, 2-arm, parallel-group, delayed-treatment, group-controlled phase 3 study | **YES**  Randomization codes were generated centrally by a contract research organization | **NO**  Open label | **NO**  Open label | **NO**  Open label | **YES**  Adherence outcome—appears to be assessed by all with 12-mo data | **YES**  No evidence of selective reporting | **PARTIAL YES**  Patients/ caregivers may have overestimated adherence (since self-reported); actual adherence may have been higher than in a real-world setting because of being part of a study |
| **Sävendahl 2020 (2)**  REAL  Multicenter, randomized, controlled, double- blind (SOMA doses only; weekly vs daily doses not blinded) phase 2 study | **YES**  Random assignment by investigators at trial sites using a trial- specific, web-based, interactive response system | **PARTIAL YES**  The three SOMA (wkly GH) doses were double-blinded; wkly vs daily GH could not be blinded | **PARTIAL YES**  The three SOMA (wkly GH) doses were double-blinded; wkly vs daily GH could not be blinded | **PARTIAL YES**  Adherence was self-reported; the 3 SOMA (weekly GH) doses were double-blinded; wkly vs daily GH could not be blinded | **PARTIAL YES**  Except for 1 of 14 patients discontinuing daily GH after 44 days, all appear to have had 52 wks of adherence data. This led to a lower mean adherence for daily GH; median values are more representative | **YES**  No evidence of selective reporting | **PARTIAL YES**  Patients/ care-givers may have overestimated adherence (since self-reported); actual adherence may have been higher than in a real-world setting because of being part of a study |

Available: <https://methods.cochrane.org/bias/resources/rob-2-revised-cochrane-risk-bias-tool-randomized-trials>

*Refer to abbreviation list on last page.*

**Table S5** | Quality assessment of observational studies using NOS.

|  | **Selection** | | | | **Comparability** | **Outcome** | | | **Total** |
| --- | --- | --- | --- | --- | --- | --- | --- | --- | --- |
| **Author, year** | **Representative-ness of exposed cohort*** | **Representative -ness of non-exposed cohort/ exposed cohort*** | **Method to ascertain exposure*** | **Outcome of interest not present at start*** | **Comparability of cohorts on basis of design or analysis**** | **Method of assessing outcome*** | **Follow-up long enough for outcomes to occur*** | **Adequacy of follow-up of cohorts*** | **Total score  (out of 9)** |
| Arrabal Vela, 2018 (3) | 0 | 0 | 1 | 1 | 0 | 1 | 1 | 1 | 5 |
| Blanco-Lopez, 2020 (5) | 1 | 0 | 1 | 1 | 0 | 1 | 1 | 1 | 6 |
| Cardinale, 2019 (6) | 1 | 0 | 1 | 1 | 0 | 1 | 0 | 0 | 4 |
| Centonze, 2019 (7) | 1 | 0 | 1 | 1 | 0 | 1 | 1 | 0 | 5 |
| Charmandari, 2020 (8) | 1 | 0 | 1 | 1 | 0 | 1 | 1 | 1 | 6 |
| De Pedro, 2016 (9) | 0 | 0 | 1 | 1 | 2 | 1 | 1 | 1 | 7 |
| Dumitrescu, 2020 (10) | 1 | 0 | 1 | 1 | 0 | 0 | 1 | 1 | 5 |
| Farfel, 2019 (11) | 1 | 0 | 1 | 1 | 1 | 1 | 1 | 1 | 7 |
| Gau, 2017 (12) | 0 | 0 | 1 | 1 | 0 | 0 | 1 | 1 | 4 |
| Ghezzi, 2017 (23) | 1 | 0 | 1 | 1 | 0 | 1 | 1 | 0 | 5 |
| Koledova, 2018 (14) | 1 | 0 | 1 | 1 | 0 | 1 | 1 | 1 | 6 |
| Koledova, 2020 (15) | 1 | 0 | 1 | 1 | 0 | 1 | 1 | 1 | 6 |
| Lass, 2015 (16) | 0 | 0 | 1 | 1 | 2 | 1 | 1 | 1 | 7 |
| Loche, 2016 (17) | 1 | 0 | 1 | 1 | 0 | 1 | 1 | 0 | 5 |
| Maggio, 2018 (18) | 0 | 0 | 1 | 1 | 1 | 1 | 1 | 0 | 5 |
| Michaelidou, 2019 (19) | 0 | 0 | 1 | 1 | 1 | 1 | 1 | 1 | 6 |
| Rodriguez Arnao, 2019 (21) | 1 | 0 | 1 | 1 | 2 | 1 | 1 | 1 | 8 |
| van Dommelen, 2018 (22) | 1 | 0 | 1 | 1 | 0 | 1 | 1 | 1 | 6 |

NOS, Newcastle–Ottawa Scale.

*Maximum of 1 point.

**Maximum of 2 points.

Score: ≥7, good; 4–6, fair; 0–3, poor.

**Table S6** | Quality assessment of survey studies using the modified NOS.

|  | **Selection** | | | | **Comparability** | **Outcome** | | **Total** |
| --- | --- | --- | --- | --- | --- | --- | --- | --- |
|  | **Representativeness of sample cohort** | **Sufficiency of sample size** | **Adequacy of response rate or data on non-responders** | **Method to ascertain exposure** | **Comparability of cohorts on basis of design or analysis** | **Method to assess outcome** | **Appropriate statistical test used** | **Total score (out of 9)** |
| **Author, year** | ***** | ***** | ***** | ***** | ****** | ****** | ***** |  |
| Bagnasco, 2017 (4) | 1 | 1 | 0 | 1 | 2 | 0 | 1 | 6 |
| Kappelgaard, 2015 (13) | 1 | 0 | 0 | 0 | 0 | 0 | 1 | 2 |
| Mohseni, 2018 (20) | 0 | 0 | 0 | 1 | 1 | 0 | 1 | 3 |

NOS, Newcastle–Ottawa Scale.

*Maximum of 1 point.

**Maximum of 2 points

Score: ≥7, good; 4-6, fair; 0-3, poor.

Source: Herzog R, et al. (24).

**Table S7** | Methods for measuring adherence and reported adherence in included studies (*n* = 23).

| **Author, year (country)** | **Population and sample size, *n* (%)** | **Adherence measurement method** | **Categorical measures of adherence** | **Reported adherence** |
| --- | --- | --- | --- | --- |
| **Interventional studies (*n* = 2)** | | | |  |
| Chung, 2018 (1) (Korea) | With 12 mo of data  ISS: 79 (100)  Tx: 52 (65.8)  *****Control: 27  (34.2) | Self-report: the proportion of prescribed doses administered according to drug diaries | Non-adherent: receipt of <75% of expected injections | Mean over 12 mo:  Treated: 93.27%  *****Control: 95.69% (over 6 m) |
| Sävendahl, 2020 (2) (11 countries) | GHD: 59 (100) | Self-report: the proportion of prescribed doses administered according to drug diaries | None reported | Mean (SD); median over 12 mo: SOMA (0.04 mg/kg/wk): 97.5% (4.50); median 99.1%  SOMA (0.08 mg/kg/wk): 98.6% (1.66);  median 100%  SOMA (0.16 mg/kg/wk): 96.3% (5.19);  median 99.1%  NORD GH (0.034 mg/kg/d):  91.8% (23.03); median 99.2% |

| **Observational studies (*n* = 21; 20 rhGH studies; 1 MS study)** | | | |  |
| --- | --- | --- | --- | --- |
| Arrabal Vela, 2018** (3) (Spain) | Total: 30  GHD: 14 (46.7)  SGA: 14 (46.7)  Other: 2 (6.6) | (Days administered at the prescribed dose/days prescribed) × 100 as measured by easypod™ injector device | Excellent adherence: >95%  Good adherence: >85–95%  Fair adherence: 75–85%  Poor adherence: <75% | Over 12 mo:  Mean (95% CI): 92.3% (87.7–96.9%)  Excellent adherence (>95%): 60%  Good adherence (>85–95%): 30%  Fair adherence (75–85%): 3.3%  Poor adherence (<75%): 6.7% |
| Bagnasco, 2017 (4) (Italy) | Total: 1007  Parents: 771  (76.6%)  Patients: 221 (21.9%)  Unknown: 15 (1.5%)  Indication: NR (all were rhGH  recipients) | Self-report (recall): the number of injections missed in a typical week over the past 12 months | Adherent: 0 missed doses in a typical week  Non-adherent: ≥1 missed  dose in a typical week | Over 12 mo:  94.5% missed ≤1 dose/wk  72.1% missed 0 doses/wk  22.4% missed 1ose/wk 2.0% missed ≥2 doses/wk 3.5% did not respond |
| Blanco-Lopez, 2020** (5) (Mexico) | Total: 147  GHD: 118 (80.3)  SGA: 24 (16.3)  TS: 5 (3.4) | (Days administered at the prescribed dose/days prescribed) × 100 as measured by easypod™ injector device | NR | Over 12 mo:  Mean 85.7%; median 92.9% |
| Cardinale, 2019** (6) (Italy) | Total: 90  GHD: 83 (92)  SGA: 5 (6)  TS: 2 (2) | (Days administered at the prescribed dose/days prescribed) × 100 as measured by easypod™ injector device | NR | Over 977 days:  mean (SD) 70% (13%) |
| Centonze, 2019** (7) (Italy) | GHD: 73 (100) | (Days administered at the prescribed dose/days prescribed) × 100 as measured by easypod™ injector device | NR directly, but >85% adherence mentioned as benchmark | Over 12 mo:  mean 88.55%; median 92.3% |
| Charmandari, 2020** (8) (Greece) | Total: 88  GHD: 78 (88.6)  SGA: 4 (4.6)  TS: 3 (3.4)  Other/missing: 3  (3.4) | (Days administered at the prescribed dose/days prescribed) × 100 as measured by easypod™ injector device | Adherent: mean adherence  rate ≥85%  (≥1 missed dose/wk on  average) | Over 12 mo:  Mean 92.5%; median (IQR) 95.5% (90.05%; 98.25%) |
| De Pedro, 2016 (9) (Spain) | Total: 158  GHD: 121 (76.5)  SGA 37 (23.5) | Annual dose prescribed compared with the doses patients picked up at the hospital pharmacy | Good: ≥92% of prescribed doses (≤2 doses missed/m) Moderate: 85 to <92%  Poor: <85% (≥1 dose  missed/wk) | Good: 66.5% of patients picked up ≥92% of their prescribed medication Moderate–good: 79% picked up ≥85% of their prescribed medication |
| Dumitrescu, 2020 (10) (Romania) | GHD: 187 (100) | Self-report (recall)  “During the last 3 mo of therapy, how often was a dose (i.e., injection) missed/skipped?” | Qualitative, 5-item Likert scale:  No missed injections  1–3 missed injections  4–6 missed injections  7–10 missed injections  >10 missed injections | In past 3 mo, % of patients who missed:  0 injections: 85.1% (95% CI: 79.4–90.7%)  1–3 injections: 8.4% (95% CI: 4.1–12.8%)  4–6 injections: 1.9% (95% CI: 0–4.1%)  7–10 injections: 0  >10 injections: 4.5% (95% CI: 1.3–7.8%) |
| Farfel, 2019 (11) (Israel) | Total: 2263 GHD, SGA, or TS (cannot differentiate): 1612 (71.2)  ISS: 575 (25.6)  CRF: 76 (3.4) | The number of months per year of pharmacy purchase of rhGH, assessed over ≥2 yr | Good: 11–12 m purchase/year Moderate: 7–10 m purchase/year  Poor: <7 m purchase/year | Over 12 mo (Year 2): mean (SD) 8.8 (2.6) mo  (calculated mean 73.3%) |
| Gau, 2017 (12) (Japan) | GHD: 46 (100) | Self-report (recall): number of missed injections in a week | Good: ≤1 missed dose/wk  Poor: >1 missed dose/wk | Over 12 mo, % who missed ≤1 dose/week  No choice of device: 95% Choice of device: 100% |
| Kappelgaard, 2015 (13) (France, Germany, Italy, USA) | Storage flexible: Total: 50† GHD/MPHD: 38 (76)  SGA: 8 (16)  TS: 4 (8)  Other: 1 (2) Refrigeration-only:  Total: 96†  GHD/MPHD: 65  (68)  SGA: 21 (22)  TS: 12 (13)  ISS: 3 (3)  RSS: 4 (4)  Other: 3 (3) | Self-report (recall): number of missing/skipped injections in a typical month | Missed no injections/mo  Missed ≥1 injection/mo | Over 12 mo:  % who missed 0 injections/month Total: 64%  Storage flexible: 76%  Refrigeration-only: 57%  % who missed ≥1 injection/month  Total: 36%  Storage flexible: 24%  Refrigeration-only: 43% |
| Koledova, 2018** (14) (24 countries) | Total: 1203  GHD: 897 (74.6)  SGA: 207 (17.2)  TS: 82 (6.8)  Other: 17 (1.4) | (Days administered at the prescribed dose/days prescribed) × 100 as measured by easypod™ injector device | NR directly, but >80% adherence mentioned as benchmark | Over 12 mo:  Total: mean 84.1%; median 93.7%  GHD: median 93.4%  SGA: median 95.0%  TS: median 93.2% |
| Koledova, 2020** (15) (multi-country) | Total: 13,553 Indication NR, all patients received rhGH | (Days administered at the prescribed dose/days prescribed) × 100 as measured by easypod™ injector device | High: ≥85% (missed ≤1 dose in a typical week) Intermediate: >56–84% Low: ≤56% | Over 12 mo:  % patients in each adherence group:  High (≥85%): 77%  Intermediate (>56–84%): 15%  Low (≤56%): 7% |
| Lass, 2015 (16) (Germany) | Total: 103  GHD: 74 (71.8)  SGA: 21 (20.4)  TS: 4 (3.9)  SHOX deficiency: 3 (2.9)  PWS: 1 (1.0) | (Doses filled at pharmacy/doses prescribed) × 100 | Good: >85.7% (<1 missed dose/wk)  Medium: 57.1–85.7% (1–3 missed doses/wk)  Poor: <57.1% (>3 missed doses/wk) | Over 12 mo: median (IQR): 91% (55–103%)§  % who missed <1 dose/wk (>85.7% adherence)  Total (*n*=103): 51%  GHD (*n*=74): 53%  SGA (*n*=21): 38%  TS (*n*=4): 75%  SHOX deficiency (*n*=3): 67%  PWS (*n*=1): 100% |
| Loche, 2016** (17) (Italy) | GHD: 79 (100) | (Days administered at the prescribed dose/days prescribed) × 100 as measured by easypod™ injector device | Fully adherent: ≥92% | Over 12 mo:  52.83% were fully (≥92%) adherent |
| Maggio, 2018** (18) (Italy) | Total: 40  GHD: 26 (65)  SGA: 9 (22.5)  TS: 5 (12.5) | (Days administered at the prescribed dose/days prescribed) × 100 as measured by easypod™ injector device | Good: ≥85% | Time period NR. mean 92.2% |
| Michaelidou, 2019 (19) (UK) | Total: 52  GHD: 34 (65.4)  TS: 5 (9.2)  Other: 13 (25.0) | Proportion of days covered: quantity of device-heads delivered × length of time each head should last [1 wk]/number of days prescribed GH treatment during treatment period | Good: >80% proportion of days covered | Over 12 mo: % with >80% proportion of days covered  Total: 57.5%  GHD: 55.9%  TS: 40%  Other: 69.2% |
| Mohseni, 2018 (20) (Iran) | Total: 169 Indication NR, all patients received rhGH | Self-reported (2 methods): (1) 8-item MMAS; (2) “auto-compliance method”: number of injections given/number of prescribed injections during the past month | MMAS: adherent = high or moderate adherence (MMAS ≥6)  High: MMAS = 8  Moderate: MMAS = 6–7.9  Low: MMAS <6  Auto-compliance:  adherent: adherence >80% | Time period NR  MMAS adherence group:  Adherent (high + moderate): 57.4% High adherence: 16.6%  Moderate adherence: 40.8%  Low adherence: 42.6%  Auto-compliance (>80% adherent): 95.3% |
| Rodriguez Arnao, 2019** (21) (Spain) | Total: 238  GHD: 144 (60.5)  SGA: 86 (36.1)  TS: 8 (3.4) | (Days administered at the prescribed dose/days prescribed) × 100 as measured by easypod™ injector device | Good: ≥85% (≤1 missed  dose/wk)  Non-adherent: <85% (>1 missed dose/wk) | Over 12 mo:  mean 95.3% (95% CI 93.3–97.2); median: 99.1 |
| van Dommelen, 2018** (22) (24 countries) | GHD (Tx-naïve): 95 (100) | (Days administered at the prescribed dose/days prescribed) × 100 as measured by easypod™ injector device | “Recursive partitioning used to find the cut-off point (the first split) for high and low adherence that maximizes the correlation between adherence and height gain”  Year 1: High: ≥98%  Year 2: ≥91%  Years 1–2: ≥78% | Over 12 mo:  Mean (SD); median (min, max)  Year 0–1: 80.8% (31.1%); 95.1% (0, 100)  Year 1–2: 81.5% (23.0%); 92.9% (0, 100) |
| **Observational studies, agents other than rhGH (*n* = 1)** | | | | |
| Ghezzi, 2017 (Italy) | MS: 40 (100) | (Number of administered injections/expected number of injections) × 100 as measured  by the RebiSmart™ device | NR | 67.5% adherence at 12 mo |

*Control consisted of 6 months without rhGH treatment, followed by 6 months with rhGH treatment. Treated received 12 months of rhGH.

**easypod™ study (includes prospective ECOS and retrospective studies).

†Respondents listed any diagnosis for which they received rhGH; sums exceed 100%.

§Adherence exceeded 100% if patients had filled more doses than prescribed.

**TABLE ABBREVIATIONS**

**CI,** confidence interval; **CRF**, chronic renal failure; **DET**, data-extraction table; **ECOS**, easypod™ Connect Observational Study; **FUTURE**, Quality of liFe in adolescent sUbjecTs affected by mUltiple sclerosis treated with immunomodulatoRy agEnt using self-injecting device; **GHD**, growth hormone disorder; **GH**, growth hormone; **IBS**, irritable bowel disease; **IQR**, interquartile range; **ISS**, idiopathic short stature; kg, kilogram; **MPHD**, multiple pituitary hormone deficiency; **MS**, multiple sclerosis; **NORD**, Norditropin; **NR**, not reported; **PC**, patient choice (of device); **PWS**, Prader–Willi syndrome; **RCT**, randomized controlled trial; **rhGH**, recombinant human growth hormone; **RoB2**, Risk of Bias tool version 2; **RSS**, Russell–Silver syndrome; **SD**, standard deviation; **SGA**, small for gestational age; **SHOX**, short stature homeobox-containing gene; **SIEDP/ISPED**, Italian Society for Pediatric Endocrinology and Diabetes; **SOMA**, somapacitan; **TS**, Turner syndrome; **Tx**, treated/treatment.

**REFERENCES**

1. Chung WY, Yoo HW, Hwang JS, Ko CW, Kim HS, Jin DK, et al. Effect of growth hormone therapy on height velocity in Korean children with idiopathic short stature: a phase III randomised controlled trial*.* Horm Res Paediatr (2018) 90:44-53. doi: 10.1159/000491016

2. Sävendahl L, Battelino T, Brod M, Hojby Rasmussen M, Horikawa R, Juul RV, et al. Once-weekly somapacitan vs daily GH in children with GH deficiency: results from a randomized phase 2 trial*.* J Clin Endocrinol Metab (2020) 105doi: 10.1210/clinem/dgz310

3. Arrabal Vela MA, Garcia Gijon CP, Pascual Martin M, Benet Gimenez I, Areas Del Aguila V, Munoz-Rodriguez JR, et al. Adherence to somatotropin treatment administered with an electronic device*.* Endocrinol Diabetes Nutr (2018) 65:314-8. doi: 10.1016/j.endinu.2018.02.003

4. Bagnasco F, Di Iorgi N, Roveda A, Gallizia A, Haupt R, Maghnie M, et al. Prevalence and correlates of adherence in children and adolescents treated with growth hormone: a multicenter Italian study*.* Endocr Pract (2017) 23:929-41. doi: 10.4158/EP171786.OR

5. Blanco-Lopez A, Antillon-Ferreira C, Saavedra-Castillo E, Barrientos-Perez M, Rivero-Escalante H, Flores-Caloca O, et al. Adherence to treatment in children with growth hormone deficiency, small for gestational age and Turner syndrome in Mexico: results of the Easypod connect observational study (ECOS)*.* J Endocrinol Invest (2020) 43:1447-52. doi: 10.1007/s40618-020-01218-4

6. Cardinale GM, Pesce S, Ingletto D, Mariano M, Catucci A, Corciulo N, et al. Monitoring of treatment adherence with easypod in six Italian centers: a real-world experience*.* Minerva Endocrinol (2019) 44:246-51. doi: 10.23736/S0391-1977.18.02843-2

7. Centonze C, Guzzetti C, Orlando G, Loche S, Italian EI. Adherence to growth hormone (GH) therapy in naive to treatment GH-deficient children: data of the Italian Cohort from the Easypod Connect Observational Study (ECOS)*.* J Endocrinol Invest (2019) 42:1241-4. doi: 10.1007/s40618-019-01046-1

8. Charmandari E, Vlachopapadopoulou E, Kyritsi EM, Sakellariou D, Koledova E, Nespithal K, et al. Adherence and long-term outcomes of therapy in paediatric patients in Greece using the easypod electromechanical device for growth hormone treatment: The phase IV multicentre easypod connect observational study (ECOS)*.* Growth Horm IGF Res (2020) 53-54:101336. doi: 10.1016/j.ghir.2020.101336

9. De Pedro S, Murillo M, Salinas I, Granada ML, Martinez M, Puig-Domingo M, et al. Variability in adherence to rhGH treatment: Socioeconomic causes and effect on children's growth*.* Growth Horm IGF Res (2016) 26:32-5. doi: 10.1016/j.ghir.2015.12.002

10. Dumitrescu CP, Procopiuc C, Dumitriu N, Micle I, Anton M, Moisuc A. COMPLIA: A 12-month prospective, multicentre, non-interventional study to evaluate treatment adherence and treatment satisfaction in a growth hormone deficient paediatric population treated with Nutropinaq® a somatropin analogue*.* Acta Endocrinol (Buchar) (2020) 16:192-8. doi: 10.4183/aeb.2020.192

11. Farfel A, Shalitin S, Morag N, Meyerovitch J. Long-term adherence to growth hormone therapy in a large health maintenance organization cohort*.* Growth Horm IGF Res (2019) 44:1-5. doi: 10.1016/j.ghir.2018.10.004

12. Gau M, Takasawa K. Initial patient choice of a growth hormone device improves child and adolescent adherence to and therapeutic effects of growth hormone replacement therapy*.* J Pediatr Endocrinol Metab (2017) 30:989-93. doi: 10.1515/jpem-2017-0146

13. Kappelgaard AM, Metzinger CP, Schnabel D. A web-based survey assessing the impact of storage flexibility on the daily life of patients and caregivers administering growth hormone*.* Expert Rev Med Devices (2015) 12:517-27. doi: 10.1586/17434440.2015.1069180

14. Koledova E, Stoyanov G, Ovbude L, Davies PSW. Adherence and long-term growth outcomes: results from the easypod() connect observational study (ECOS) in paediatric patients with growth disorders*.* Endocr Connect (2018) 7:914-23. doi: 10.1530/EC-18-0172

15. Koledova E, Tornincasa V, van Dommelen P. Analysis of real-world data on growth hormone therapy adherence using a connected injection device*.* BMC Med Inform Decis Mak (2020) 20:176. doi: 10.1186/s12911-020-01183-1

16. Lass N, Reinehr T. Low treatment adherence in pubertal children treated with thyroxin or growth hormone*.* Horm Res Paediatr (2015) 84:240-7. doi: 10.1159/000437305

17. Loche S, Salerno M, Garofalo P, Cardinale GM, Licenziati MR, Citro G, et al. Adherence in children with growth hormone deficiency treated with r-hGH and the easypod device*.* J Endocrinol Invest (2016) 39:1419-24. doi: 10.1007/s40618-016-0510-0

18. Maggio MC, Vergara B, Porcelli P, Corsello G. Improvement of treatment adherence with growth hormone by easypod device: experience of an Italian centre*.* Ital J Pediatr (2018) 44:113. doi: 10.1186/s13052-018-0548-z

19. Michaelidou M, Whitten S, Bajaj P, Knight A, Spoudeas HA. Improved adherence and growth outcomes with jet-delivered growth hormone*.* J Pediatr Endocrinol Metab (2019) 32:207-13. doi: 10.1515/jpem-2018-0067

20. Mohseni S, Heydari Z, Qorbani M, Radfar M. Adherence to growth hormone therapy in children and its potential barriers*.* J Pediatr Endocrinol Metab (2018) 31:13-20. doi: 10.1515/jpem-2017-0157

21. Rodriguez Arnao MD, Rodriguez Sanchez A, Diez Lopez I, Ramirez Fernandez J, Bermudez de la Vega JA, Yeste Fernandez D, et al. Adherence and long-term outcomes of growth hormone therapy with easypod in pediatric subjects: Spanish ECOS study*.* Endocr Connect (2019) 8:1240-9. doi: 10.1530/EC-19-0325

22. van Dommelen P, Koledova E, Wit JM. Effect of adherence to growth hormone treatment on 0-2 year catch-up growth in children with growth hormone deficiency*.* PLoS One (2018) 13:e0206009. doi: 10.1371/journal.pone.0206009

23. Ghezzi A, Bianchi A, Baroncini D, Bertolotto A, Malucchi S, Bresciamorra V, et al. A multicenter, observational, prospective study of self- and parent-reported quality of life in adolescent multiple sclerosis patients self-administering interferon-beta1a using RebiSmart-the FUTURE study*.* Neurol Sci (2017) 38:1999-2005. doi: 10.1007/s10072-017-3091-6

24. Herzog R, Alvarez-Pasquin MJ, Diaz C, Del Barrio JL, Estrada JM, Gil A. Are healthcare workers' intentions to vaccinate related to their knowledge, beliefs and attitudes? A systematic review*.* BMC Public Health (2013) 13:154. doi: 10.1186/1471-2458-13-154
